# The Number of Newly Dispensed Hypnotic Drugs During the First COVID‐19 Lockdown Period in The Netherlands

**DOI:** 10.1111/jsr.70190

**Published:** 2025-08-25

**Authors:** Dana M. Dijkgraaf, Pantea Kiani, Maureen N. Zijlstra, Pauline A. Hendriksen, Johan Garssen, Joris C. Verster

**Affiliations:** ^1^ Division of Pharmacology, Utrecht Institute for Pharmaceutical Sciences Utrecht University Utrecht the Netherlands; ^2^ Danone Global Research and Innovation Center Utrecht the Netherlands; ^3^ Cognitive Neurophysiology, Department of Child and Adolescent Psychiatry, Faculty of Medicine TU Dresden Dresden Germany; ^4^ Centre for Mental Health and Brain Sciences Swinburne University Melbourne Australia

**Keywords:** COVID‐19, delayed healthcare, hypnotic drugs, lockdown, prescriptions

## Abstract

The 2019 coronavirus (COVID‐19) pandemic and associated lockdowns significantly disrupted healthcare systems, including access to pharmacological treatments such as sleep medication. This study investigated the number of first‐time dispensed hypnotic drugs during the first COVID‐19 lockdown in the Netherlands, using data from the Dutch Foundation for Pharmaceutical Statistics (SFK), which covers approximately 96% of all pharmacy dispensations (5.46 million patients). First‐time dispensing was defined as no use of hypnotics in the previous year and included benzodiazepines, benzodiazepine‐related drugs, and melatonin receptor agonists. Data from 2020 were analysed across three periods: pre‐lockdown (Weeks 1–11), lockdown (Weeks 12–19) and post‐lockdown (Weeks 20–26), and were compared to the same periods in 2019. Analyses were stratified by age group (children: 0–9, adolescents: 10–19, adults: 20–64, elderly: 65+) and sex. The data revealed a significant reduction in first‐time hypnotic dispensations in 2020 (155,961) compared to 2019 (168,814, *p* < 0.001), with declines across all three time periods (*p* < 0.001). During the lockdown, significant reductions were found among children, adolescents, and adults (*p* < 0.001), but not among the elderly. Female adults and the elderly received significantly more hypnotics than their male counterparts (*p* < 0.001), consistent with higher reported rates of sleep disturbances. In conclusion, the overall number of first‐time dispensed hypnotics was significantly lower during the first COVID‐19 lockdown in the Netherlands, except among the elderly. It remains unclear to what extent individuals self‐medicated with over‐the‐counter sleep medication, or experienced untreated sleep complaints during the first lockdown in the Netherlands.

## 1 Introduction

The 2019 coronavirus disease (COVID‐19) pandemic significantly disrupted daily life across the globe. In the Netherlands, the first national lockdown period—from 15 March 2020 to 11 May 2020—required people to work from home and limit physical social interactions to reduce the spread of the SARS‐CoV‐2 virus (Rijksoverheid 2020a). While non‐essential venues such as shops, restaurants, and social venues were closed, essential services such as supermarkets and pharmacies remained operational. At that time, no vaccines or effective treatments were available. The fear of infection and stay‐at‐home restrictions had a substantial impact on mental well‐being, contributing to elevated levels of anxiety, stress, depression, and loneliness (Kiani et al. 2023; Penninx et al. 2022). These effects were mirrored in physical health outcomes, including impaired immune fitness and increased reports of sleep disturbances (Hendriksen et al. 2021; Limongi et al. 2023; Grigsby et al. 2021).

The Dutch healthcare system was also significantly affected, experiencing both increased workloads for healthcare professionals and widespread delays in care (De Vroege and van den Broek 2022; Visscher et al. 2023). Studies suggest that approximately 31% of Dutch citizens encountered postponed healthcare during the pandemic, with 14% of delays initiated by healthcare providers, 12% by patients and 5% involving both parties (Visscher et al. 2023). Physical visits to general practitioners dropped by 11% during the first lockdown, while electronic consultations increased (Rijksinstituut voor Volksgezondheid en Milieu (RIVM) 2021). Postponements were often driven by fear of infection when encountering other patients and the reallocation of healthcare resources to COVID‐19 care (Rijksinstituut voor Volksgezondheid en Milieu (RIVM) 2021)—factors that likely affected prescription rates, including first‐time prescriptions for hypnotic medications.

Evidence on how lockdown periods affected sleep is mixed. Some individuals reported improved sleep quality, while others experienced increased sleep complaints (Kocevska et al. 2020). Reported changes varied across countries and cohorts, influenced by demographic factors such as sex and age, mental health status, and individuals' capacity to adapt to altered lifestyles (Kocevska et al. 2020; Gupta et al. 2020; Luijten et al. 2020). Children and adolescents reported worse mental health and sleep‐related impairment during lockdowns (Luijten et al. 2020). Among adults, changes included shifted sleep schedules, reduced nighttime sleep, and increased daytime napping (Gupta et al. 2020). While women are generally more likely than men to report insomnia, findings on sex differences during stressful situations such as lockdowns remain inconsistent (Pajėdienė et al. 2024; Ruiz‐Herrera et al. 2023; Alimoradi et al. 2021). Disrupted sleep–wake rhythms due to the absence of structured work routines may have worsened sleep for some, whereas others may have benefited from the opportunity to rest when needed. Given this ambiguity, it remains unclear to what extent the lockdown's impact on sleep translated into increased or decreased use of hypnotic drugs.

Studies from France (Beck et al. 2021), Scandinavia (Cesta 2023), and the USA (Grigsby et al. 2021) reported increased sleep medication use during the first lockdown. However, research from Northern Ireland found that after an initial spike in prescriptions—likely due to stockpiling in March 2020—usage quickly normalised (Maguire et al. 2022). In the Netherlands, a study among 350 pharmacies observed an initial rise in benzodiazepine prescriptions (including both anxiolytics and hypnotics) during week 12 of 2020, followed by a return to baseline levels compared to 2019 (Hek et al. 2020). However, that study had several limitations: it covered only a subset of pharmacies, did not distinguish hypnotics from other benzodiazepines, and similar to the French and Northern Irish studies included all users of benzodiazepine drugs. Another Dutch study evaluated the use of benzodiazepines during the COVID‐19 pandemic (Visser et al. 2025). They reported no change in the number of first‐time users of benzodiazepine drugs during the first lockdown period in the Netherlands. This study also did not differentiate between the use for hypnotic or anxiolytic purposes. In addition, all non‐benzodiazepine hypnotic drugs were excluded from these analyses. Finally, next to possible sleep disturbances, literature revealed a significant fear of COVID‐19, in particular during the first lockdown period (Chan et al. 2024). As this may have influenced first‐time prescriptions of benzodiazepine anxiolytic drugs, the combined presentation of effects of all benzodiazepine drugs is less informative.

As none of the previous studies specifically focused on first‐time users of hypnotic drugs, the aim of the current study was to evaluate drugs that are more commonly associated with sleep disturbances. Only first‐time dispensed hypnotic drugs during the first lockdown period in the Netherlands were analysed. These included all types of hypnotics prescribed in the Netherlands—such as benzodiazepines, Z‐drugs, melatonin receptor agonists, and orexin receptor antagonists. Data were obtained from the Dutch Foundation for Pharmaceutical Statistics (SFK), which encompasses 96% of pharmacy dispensations nationwide (Stichting Farmaceutische Kerngetallen 2023). The data were analysed for the first half of 2020—including the lockdown period—and compared to the same period in 2019. A second aim of the study was to analyse the data stratified by age and sex. Previous studies have not reported on possible sex and age differences, while the literature suggests that there are significant differences in these groups regarding the incidence of sleep complaints (Pajėdienė et al. 2024).

It can be hypothesised that the COVID‐19 lockdown period is associated with an increase in sleep disturbances, which may be reflected in increased first‐time hypnotic drug use. However, there are documented delays in healthcare during the pandemic (Visscher et al. 2023; Rijksinstituut voor Volksgezondheid en Milieu (RIVM) 2020), which may reduce the number of first‐time dispensed hypnotic drugs. Therefore, it was hypothesised that the first COVID‐19 lockdown would be associated with a significant reduction in the number of first‐time dispensed hypnotic drugs across all age groups and for both sexes.

## 2 Methods

SFK provided data on the number of first‐time dispensed hypnotic drugs, i.e., hypnotic drugs dispensed to new patients who received their hypnotic drug for the first time during the first half of 2019 or the first half of 2020. First‐time use was defined as having no recorded dispensing of a hypnotic drug in the 12 months preceding the prescription date.

The analysis included all hypnotic drugs classified under ATC3 N05C of the Anatomical Therapeutic Chemical Classification System by the World Health Organisation (World Health Organization Collaborating Centre for Drug Statistics Methodology 2023). These include benzodiazepines (e.g., temazepam and nitrazepam), benzodiazepine‐related drugs (e.g., zopiclone and zolpidem), melatonin receptor agonists (e.g., melatonin and ramelteon), orexin receptor antagonists (e.g., suvorexant), barbiturates (e.g., pentobarbital and secobarbital), aldehydes (e.g., chloral hydrate), piperidinedione derivatives (e.g., glutethimide) and others (e.g., clomethiazole). Over‐the‐counter (OTC) sleep aids were not included in the dataset.

The dataset covered 1890 Dutch pharmacies, representing approximately 96% of the Dutch population (5.46 million patients). Collected patient information included sex (male or female) and age group: children (0–9 years old), adolescents (10–19 years old), adults (20–64 years old) and the elderly (65 year and older). In addition, the date of first‐time dispensing was categorised by week number (Week 1–Week 26). The Dutch government installed a lockdown period covering weeks 12–19 of 2020 (Rijksoverheid 2020b). During this period, starting on 15 March 2020, several measures were installed to spread the SARS‐CoV‐2 virus. Most importantly were keeping 1.5 m distance and the closure of schools and nonessential businesses. Most of these measures were lifted in week 19 of 2020 (Rijksoverheid 2020c). To examine the impact of this first national COVID‐19 lockdown, the data from 2020 were divided into three time periods, including (1) pre‐lockdown: weeks 1–11, (2) lockdown: weeks 12–19 and (3) post‐lockdown: weeks 20–26. These were compared to the same calendar weeks in 2019.

### 2.1 Statistical Analysis

All Statistical analyses were performed using IBM SPSS Statistics for Mac, Version 30.0 (IBM Corp., Armonk, NY, USA). A general linear model (GLM) for repeated measures was used to compare the three time periods, with Bonferroni correction for multiple comparisons (two‐sided, *p* < 0.025 considered significant). Weekly counts of first‐time hypnotic dispensing between 2019 and 2020 were compared using paired *t*‐tests for each of the three periods. Statistical significance was set at *p* < 0.05 (two‐sided) for these comparisons. Analyses were conducted overall, as well as stratified by sex and age group.

## 3 Results

**FIGURE 1:**
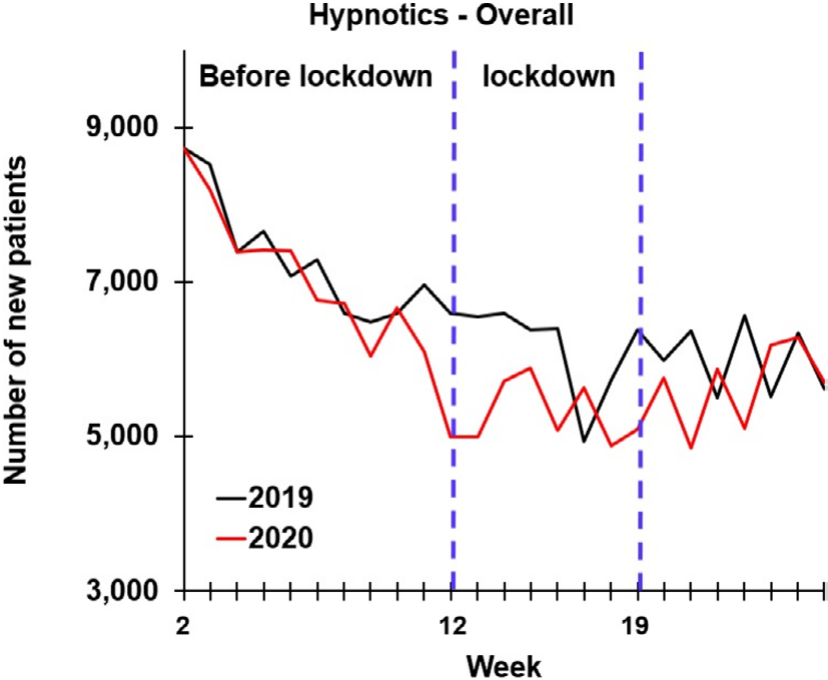
Number of newly dispensed hypnotic drugs per week in 2019 and 2020.

In the first half of both 2019 and 2020, prescription drugs were dispensed to approximately 5.46 million individuals living in the Netherlands. This analysis focused on patients who received hypnotic drugs for the first time. A summary of newly prescribed hypnotic drug data is presented in Table 1 and Figure 1.

**TABLE 1:** Total number of first‐time dispensed hypnotic drugs in the Netherlands.

Age group	Population	Year	Week 1–11	Week 12–19	Week 20–26
Overall	Overall	2019	77,320	49,587	41,907
		2020	73,607	42,320	39,870
		T	1.951	3.448	0.941
		*p*‐value	0.080	0.011*	0.383
	Male	2019	31,602	19,952	16,954
		2020	30,091	17,334	16,168
		T	2.227	3.232	0.945
		*p*‐value	0.050	0.014*	0.381
	Female	2019	45,718	29,635	24,953
		2020	43,771	24,950	27,173
		T	1.719	3.431	0.936
		*p*‐value	0.116	0.011*	0.385
Children	Overall	2019	1258	797	746
		2020	993	504	542
		T	2.855	6.355	3.562
		*p*‐value	0.017*	< 0.001*	0.012*
	Male	2019	747	497	454
		2020	782	278	286
		T	−0.565	11.454	3.974
		*p*‐value	0.584	< 0.001*	0.007*
	Female	2019	511	300	292
		2020	466	190	156
		T	1.165	3.965	7.843
		*p*‐value	0.271	0.005*	< 0.001*
Adolescents	Overall	2019	2348	1547	1335
		2020	1876	929	965
		T	5.788	5.169	4.728
		*p*‐value	< 0.001*	0.001*	0.003*
	Male	2019	1121	772	656
		2020	930	471	473
		T	3.314	4.803	3.953
		*p*‐value	0.008*	0.002*	0.008*
	Female	2019	1227	775	679
		2020	946	458	492
		T	5.990	4.887	4.063
		*p*‐value	< 0.001*	0.002*	0.007*
Adults	Overall	2019	46,737	29,521	25,061
		2020	44,435	23,334	23,278
		T	2.014	4.000	1.158
		*p*‐value	0.072	0.005*	0.291
	Male	2019	18,479	11,495	9799
		2020	17,429	8966	9081
		T	2.343	4.167	1.186
		*p*‐value	0.041	0.004*	0.280
	Female	2019	28,258	18,026	15,262
		2020	27,006	14,368	14,197
		T	1.766	3.758	1.131
		*p*‐value	0.108	0.007*	0.301
Elderly	Overall	2019	26,977	17,722	14,765
		2020	26,303	17,553	15,085
		T	1.030	0.235	−0.478
		*p*‐value	0.327	0.821	0.650
	Male	2019	11,255	7188	6045
		2020	10,950	7619	6328
		T	1.269	−1.311	−1.132
		*p*‐value	0.233	0.231	0.301
	Female	2019	15,722	10,534	8720
		2020	15,353	9934	8757
		T	0.803	1.389	−0.074
		*p*‐value	0.440	0.207	0.944

*Note*: Significant differences between 2019 and 2020 (*p* < 0.05) are indicated by *.

In total, 155,961 first‐time hypnotic prescriptions were dispensed in the first half of 2020—a statistically significant decrease compared to 168,814 in the same period of 2019 (T = 3.480, *p* = 0.002). The reduction was significant for the lockdown period (T = 3.448, *p* = 0.011), but not for the pre‐lockdown period (T = 1.951, *p* = 0.080) and post‐lockdown period (T = 0.941, *p* = 0.383).

In both 2019 (F = 32.692, *p* < 0.001) and 2020 (F = 37.719, *p* < 0.001), a significant downward trend was observed across the three periods. In 2019, compared to pre‐lockdown, the number of first‐time hypnotic prescriptions dropped significantly during lockdown (F = 64.090, *p* < 0.001) and post‐lockdown (F = 60.529, *p* < 0.001). In 2020, compared to pre‐lockdown, the number of first‐time hypnotic prescriptions dropped significantly during lockdown (F = 53.037, *p* < 0.001) and post‐lockdown (F = 67.997, *p* < 0.001), as illustrated in Figure 1.

**FIGURE 2:**
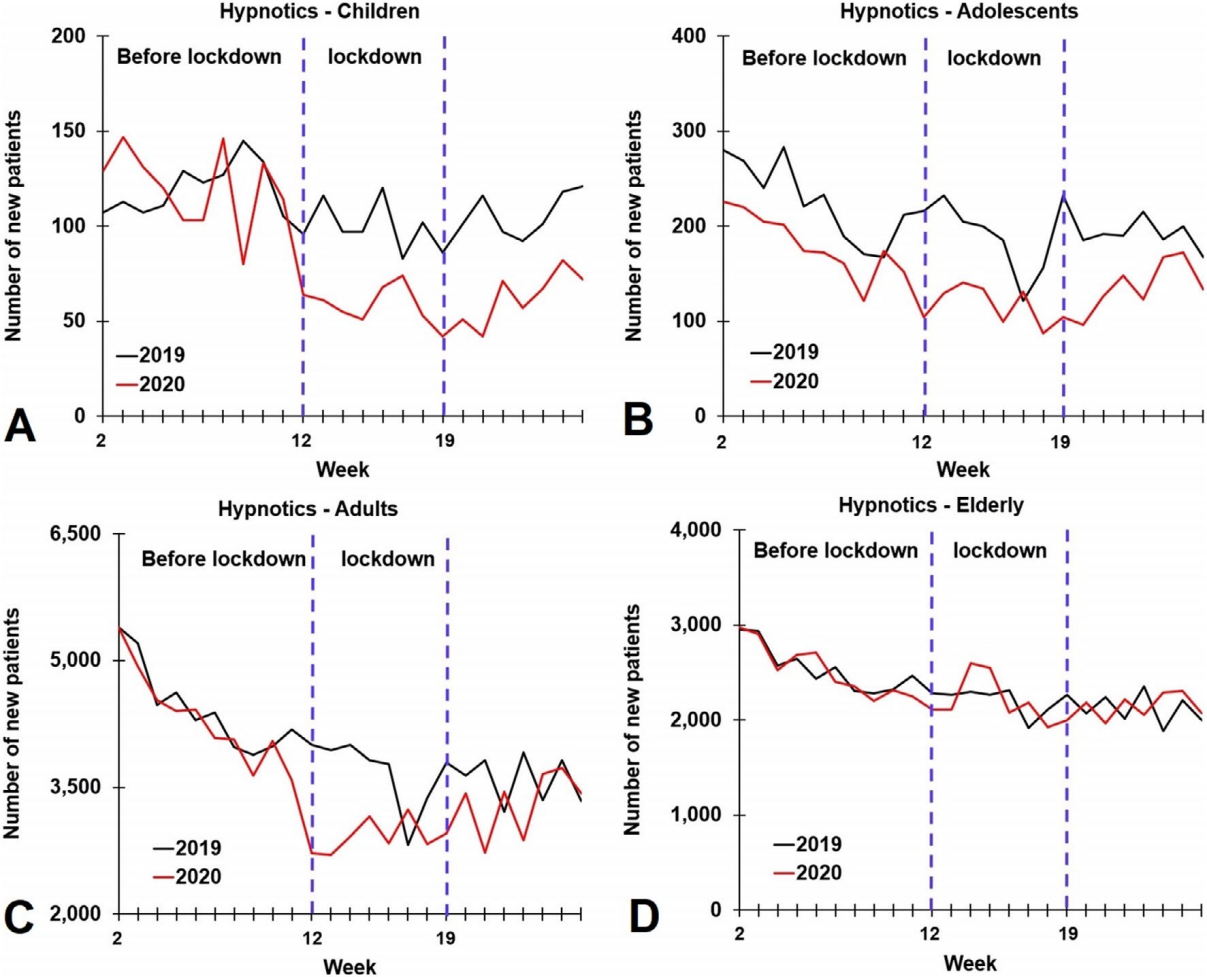
Number of newly dispensed hypnotic drugs per week in 2019 and 2020 according to age group. Data are shown according to age group. Figure (A) children, (B) adolescents, (C) adults and (D) the elderly.

Across all periods, adults received the most hypnotic prescriptions, followed by the elderly, adolescents, and children. Among children and adolescents, no significant sex differences were observed. However, among adults and the elderly, across all periods in 2019 (T = −30.625, *p* < 0.001) and 2020 (T = −19.461, *p* < 0.001), females received significantly more hypnotic prescriptions than males. A breakdown by age is shown in Figure 2.

For the pre‐lockdown period, no significant differences in the number of first‐time dispensed hypnotic drugs were observed between 2019 and 2020 for adults and the elderly. However, among children (overall) and adolescents (overall, and stratified by sex), during the pre‐lockdown period significantly fewer hypnotic drugs were dispensed in 2020 compared to 2019.

During lockdown, a significant reduction in first‐time dispensed hypnotic drugs was found in 2020 compared to 2019 for all age groups, except the elderly (See Table 1). The reduction was significant for both males and females (See Table 1).

Post‐lockdown, no significant differences were observed for adults and the elderly, whereas among children and adolescents (overall and stratified by sex), significantly fewer hypnotic drugs were dispensed in 2020 compared to 2019.

## 4 Discussion

This study found that the overall number of first‐time dispensed hypnotics was significantly lower during the first COVID‐19 lockdown in the Netherlands (2020) compared to the same period in 2019. The findings highlight distinct differences across age groups and between sexes. Specifically, adults and elderly patients received significantly more hypnotic prescriptions if they were female, consistent with prior studies reporting higher rates of sleep disturbances in women (Grigsby et al. 2021; Hek et al. 2020).

Age‐stratified analyses showed that the decrease in first‐time hypnotic use during the lockdown was significant for children, adolescents, and adults, but not for the elderly. This is notable, given that the lockdown was associated with widespread reports of increased sleep complaints, often linked to elevated anxiety, stress, and fear of infection (Kiani et al. 2023; Limongi et al. 2023; Rijksinstituut voor Volksgezondheid en Milieu (RIVM) 2020). The reduction in newly initiated hypnotic use suggests that delayed access to healthcare—rather than a decrease in sleep problems—likely drove the observed decline. Interestingly, the elderly did not show a significant decrease in first‐time hypnotic use, despite a reported 7% drop in consultations compared to 2019. This may indicate that general practitioners continued to prioritise care for vulnerable populations such as the elderly, potentially through increased use of remote consultations (Rijksinstituut voor Volksgezondheid en Milieu (RIVM) 2021).

To our best knowledge, there is no Dutch or international study published that evaluated the number of first‐time dispensed hypnotics during the first COVID‐19 wave in the Netherlands. However, several international studies that examined total prescription rates, including existing users, reported increased sleep medication use during the first lockdown (Grigsby et al. 2021; Beck et al. 2021; Cesta 2023; Maguire et al. 2022). Dutch studies looking at benzodiazepine prescriptions (including both anxiolytics and hypnotics) in all existing users also reported an increase in prescriptions during the first Dutch lockdown period (Hek et al. 2020; Visser et al. 2025). In contrast, our findings in first‐time users show a significant decrease in dispensed hypnotics across all age groups, except the elderly.

The differences between the studies can probably be explained by the difference between first‐time and existing users. Unlike studies on existing users of benzodiazepines and other medicines (Hek et al. 2020; Tiger et al. 2024), hoarding or stockpiling cannot impact the number of dispensed drugs to first‐time users. As such, this did not play a role in the current study. Previous research noted spikes in prescription volumes during the initial weeks of the COVID‐19 pandemic, likely reflecting concerns about pharmacy closures. However, those studies focused on existing users, whereas the present study exclusively examined first‐time users, providing a clearer picture of new treatment initiation for sleep disturbances during the lockdown. Lockdowns have been associated with initiating sleep problems. The current study on first‐time dispensed hypnotics better describes the possible impact of a lockdown period on sleep than datasets that also include existing users of hypnotic drugs.

Strengths of this study include the stratification by age and sex, the use of pre‐COVID‐19 data for comparison, and the focus on first‐time dispensing rates. Focusing on dispensing rather than prescription data provides a more accurate measure of actual use, as prescriptions reflect what is ordered by physicians, whereas dispensing data capture what patients actually collected. The SFK database lists the type of dispensed drugs, but not the corresponding medical diagnosis. According to the World Health Organisation, the Anatomical Therapeutic Chemical Classification System categorised medicines according to their main therapeutic use (World Health Organization, n.d.). Therefore, only medicines that are listed as hypnotic drugs in ATC3 group N05C were included in the current study. Of note, some drugs are prescribed for hypnotic purposes next to their primary intended use (e.g., the anxiolytic drug oxazepam may also be prescribed for insomnia). Drugs that are not listed in ATC3 group N05C were not included in the current analyses, as the purpose of their use could not be verified from the SFK database. Future studies should link prescription data to the corresponding diagnosis to provide more detailed information and enable the inclusion of drugs that are used for hypnotic purposes that are not listed in ATC3 group N05C, such as oxazepam. Several limitations should also be acknowledged. By including drugs from the ATC3 group N05C, some drugs that are not commonly used for sleep disturbances (i.e., midazolam and barbiturates) are included in the analysis. As no data were collected at the individual drug level, it is not known to what extent the results are driven by specific hypnotic drugs.

The analysis did not account for OTC sleep aids, such as melatonin or valerian. It remains unclear whether people with sleep complaints turned to self‐medication, or simply went untreated. Obtaining this information is critical in terms of pandemic preparedness. If indeed a substantial number of patients with sleep complaints remained untreated, policymakers should reconsider their strategies to fight viruses in future pandemics, including the development of plans to prevent delayed healthcare. Future studies should therefore explore the use of OTC sleep medications during the pandemic, as well as assess subsequent lockdowns and non‐lockdown periods to evaluate longer‐term trends in hypnotic drug use and healthcare access. Finally, research should also evaluate the effects of subsequent lockdown periods on first‐time prescriptions of hypnotic drugs.

In conclusion, the first COVID‐19 lockdown in the Netherlands was associated with a significant reduction in first‐time hypnotic dispensing for children, adolescents, and adults—possibly driven by delayed healthcare access or other factors. In contrast, prescription rates for the elderly remained stable, possibly due to sustained medical follow‐up. Women in the adult and elderly groups were more frequently prescribed hypnotics than men, a finding which is in line with the known sex‐based differences in sleep disturbance prevalence.

## Author Contributions

**Dana M. Dijkgraaf:** conceptualization, writing – original draft, writing – review and editing. **Pantea Kiani:** conceptualization, writing – review and editing. **Maureen N. Zijlstra:** conceptualization, writing – review and editing. **Pauline A. Hendriksen:** conceptualization, writing – review and editing. **Johan Garssen:** conceptualization, writing – review and editing. **Joris C. Verster:** conceptualization, methodology, formal analysis, writing – review and editing.

## Disclosure

Over the past 36 months, J.C.V. has received research grants from Danone and Inbiose and has acted as a consultant/expert advisor to Eisai, KNMP, Med Solutions, Mozand, Red Bull, Sen‐Jam Pharmaceutical, and Toast!. J.C.V., D.M.D. and M.N.Z. have received travel support from Sen‐Jam Pharmaceutical. J.C.V. owns stock from Sen‐Jam Pharmaceutical. J.G. is a part‐time employee of Nutricia Research and received research grants from Nutricia Research Foundation, Top Institute Pharma, Top Institute Food and Nutrition, GSK, STW, NWO, Friesland Campina, CCC, Raak‐Pro, and EU. P.K. is the CEO of Pangenix. P.A.H. has nothing to declare.

## Consent

The authors have nothing to report.

## Conflicts of Interest

The authors declare no conflicts of interest.

## Data Availability

The data that support the findings of this study are available from Stichting Farmaceutische Kentallen. Restrictions apply to the availability of these data, which were used under license for this study. Data are available from the author(s) with the permission of Stichting Farmaceutische Kentallen.
